# A quasi-quantitative dual multiplexed immunoblot method to simultaneously analyze ATM and H2AX Phosphorylation in human peripheral blood mononuclear cells

**DOI:** 10.18632/oncoscience.162

**Published:** 2015-05-14

**Authors:** Christopher J. Bakkenist, R. Kenneth Czambel, Pamela A. Hershberger, Hussein Tawbi, Jan H. Beumer, John C. Schmitz

**Affiliations:** ^1^ Departments of Radiation Oncology; ^2^ Pharmacology and Chemical Biology; ^3^ Medicine, University of Pittsburgh School of Medicine, 5117 Centre Avenue, Pittsburgh, PA 15213-1863; ^4^ Department of Pharmacology and Therapeutics, Roswell Park Cancer Institute, Elm & Carlton Streets, Buffalo, NY 14263; ^5^ Department of Pharmaceutical Sciences, University of Pittsburgh School of Pharmacy, 5117 Centre Avenue, Pittsburgh, PA 15213-1863

**Keywords:** DNA damage response, ATM, H2AX, doxorubicin, PBMCs

## Abstract

Pharmacologic inhibition of DNA repair may increase the efficacy of many cytotoxic cancer agents. Inhibitors of DNA repair enzymes including APE1, ATM, ATR, DNA-PK and PARP have been developed and the PARP inhibitor olaparib is the first-in-class approved in Europe and the USA for the treatment of advanced BRCA-mutated ovarian cancer. Sensitive pharmacodynamic (PD) biomarkers are needed to further evaluate the efficacy of inhibitors of DNA repair enzymes in clinical trials. ATM is a protein kinase that mediates cell-cycle checkpoint activation and DNA double-strand break repair. ATM kinase activation at DNA double-strand breaks (DSBs) is associated with intermolecular autophosphorylation on serine-1981. Exquisite sensitivity and high stoichiometry as well as facile extraction suggest that ATM serine-1981 phosphorylation may be a highly dynamic PD biomarker for both ATM kinase inhibitors and radiation- and chemotherapy-induced DSBs. Here we report the pre-clinical analytical validation and fit-for-purpose biomarker method validation of a quasi-quantitative dual multiplexed immunoblot method to simultaneously analyze ATM and H2AX phosphorylation in human peripheral blood mononuclear cells (PBMCs). We explore the dynamics of these phosphorylations in PBMCs exposed to chemotherapeutic agents and DNA repair inhibitors *in vitro*, and show that ATM serine-1981 phosphorylation is increased in PBMCs in sarcoma patients treated with DNA damaging chemotherapy.

## INTRODUCTION

Selective inhibition of DNA repair may increase the efficacy of chemotherapy regimens that include a DNA damaging modality. To this end, pharmacologic inhibitors of the apurinic/apyrimidinic endonuclease-1 (APE1) and poly(ADP-ribose) polymerase (PARP), enzymes essential for base excision repair (BER), ataxia telangiectasia mutated (ATM) and ataxia telangiectasia and Rad3-related (ATR), DNA damage signaling kinases that mediate homologous recombination repair, and DNA-dependent protein kinase (DNA-PK), whose activity is essential for non-homologous end joining (NHEJ), have been developed [1-8]. Since PARP inhibitors have antitumor activity in BRCA-deficient cancers there is considerable interest in advancing other pharmacologic inhibitors of DNA repair pathways into clinical trials [9]. The successful implementation of such trials requires the development of pharmacodynamic (PD) biomarkers for both target engagement and chemotherapy- induced DNA damage and the modulation thereof by the DNA repair modulator under investigation.

Microscopic γH2AX foci are widely used as a PD biomarker for DNA double-strand breaks (DSBs) in cells. Histone H2AX is phosphorylated on serine-139 to generate the histone derivative γH2AX in ~30 Mb of chromatin on each side of a programmed or damage-induced DSB including those associated with DNA fragmentation and apoptosis [10-12]. H2AX phosphorylation is not restricted to DSBs, however, as pan-nuclear γH2AX is induced by ultraviolet radiation (UV) and high energy β-particles [13,14]. The mechanistic significance of pan-nuclear γH2AX is not known.

ATM kinase is activated at DSBs through intermolecular autophosphorylation on serine-1981 and dimer dissociation [15]. ATM serine-1981 phosphorylation is increased in cells exposed to as little as 5 cGy γ-rays and over 50% of ATM protein is phosphorylated on this site 30 min after exposure to 0.5 Gy γ-rays [15,16]. This sensitivity and stoichiometry suggest that ATM serine-1981 phosphorylation may be an excellent PD biomarker for ATM kinase inhibitors and radiation- and chemotherapy-induced DNA damage. Since ATR also phosphorylates ATM serine-1981 in cells exposed to DNA damaging modalities that induce stalled replication forks, including UV and hydroxyurea (HU), ATM serine-1981 phosphorylation, like γH2AX, cannot be considered an unambiguous PD biomarker of DSBs [17].

We have explored the utility of ATM serine-1981 phosphorylation as a novel PD biomarker for phase I clinical trials. Here we document the pre-clinical analytical validation and fit-for-purpose biomarker validation of a quasi-quantitative dual multiplexed immunoblot method to simultaneously analyze ATM and H2AX phosphorylation in human peripheral blood mononuclear cells (PBMCs). The quasi-quantitative assay does not employ a calibration standard, but has a continuous response that can be expressed in terms of the metrics that are characteristic of the test sample [18]. We describe the dynamics of these phosphorylations in PBMCs exposed to chemotherapeutic agents and DNA repair inhibitors *in vitro,* and show that ATM serine-1981 phosphorylation is increased in PBMCs in sarcoma patients treated with doxorubicin *in vivo*. We conclude that ATM serine-1981 phosphorylation is an analytically sensitive, specific and robust PD biomarker for ATM kinase inhibitor target engagement and radiation- and chemotherapy-induced DNA damage that has a greater dynamic range than γH2AX.

## RESULTS

### Multiplexed ATM and H2AX antibody compatibility assessment

To evaluate binding interference arising within the primary antibody pairs, a series of protein extracts prepared from control PBMCs or PBMCs irradiated in whole blood were resolved in triplicate on a single gel. After resolution and electroelution transfer, the membrane was cut into triplicate blots and probed for either ATM or H2AX proteins. Since ATM and H2AX are 3056 and 141 amino acids in size, respectively, they are readily resolved by gel electrophoresis. The three blots were incubated with anti-phospho- serine-1981 ATM, anti-pan-ATM or both ATM antibodies, or anti-phospho-serine-139 H2AX, anti-pan-H2AX or both H2AX antibodies. The blots were subsequently processed in an identical manner and the effect of signal intensity caused by multiplexed antibody pairs was determined. Antibodies described in the Materials and Methods section were selected on the basis of prior experience.

**Figure 1:**
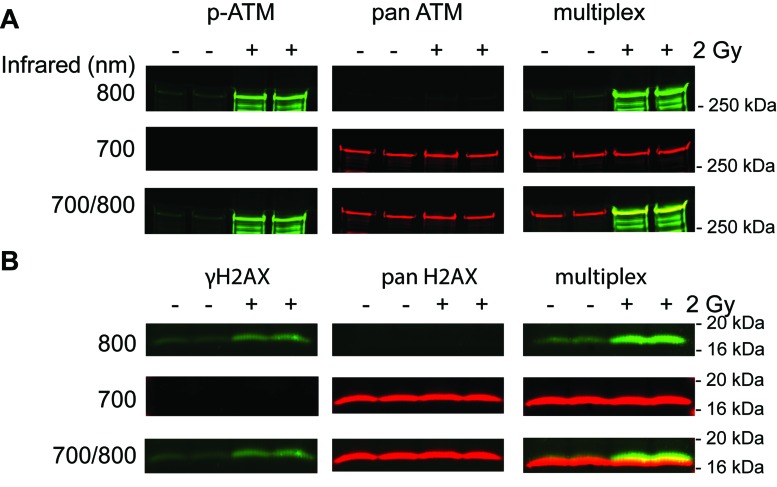
Effect of singleplex and multiplex antibody combinations on ATM (A) and H2AX (B) protein detection Whole blood was irradiated with 2 Gy and PBMCs were isolated and processed for immunoblot analysis. Primary antibodies were added separately and in combination and indicated proteins were detected at 700 and 800 nm. The blots are representative images for one of three experiments.

The ATM serine-1981 phosphorylation signal intensity was increased by 15% and 5% for the control and 2 Gy specimens, respectively, when blots were incubated in multiplexed ATM antibodies rather than the singleplexed ATM serine-1981 phosphorylation specific antibody (Figure 1A). Importantly, pan-ATM immunoblot signal intensity showed relatively little change in multiplexed ATM antibodies (−0.5% and 0.3% for the control and 2 Gy specimens, respectively). The ATM serine-1981 phosphorylation to pan-ATM signal ratio determined with the multiplexed ATM antibodies was 12% less than that determined with the singleplexed antibodies for the 2 Gy specimen.

The γH2AX signal intensity was increased by 7% and 16% for the control and 2 Gy specimens, respectively, when blots were incubated in multiplexed H2AX antibodies rather than the singleplexed γH2AX specific antibody (Figure 1B). Importantly, pan-H2AX immunoblot signal intensity showed relatively little change in multiplexed H2AX antibodies (+2% and −4% for the control and 2 Gy specimens, respectively). The γH2AX to pan-H2AX signal ratio determined with the multiplexed was 5% greater than that determined with the singleplexed antibodies for the 2 Gy specimen. Thus, combining phospho-specific and pan antibodies demonstrated little to no interference with regards to signal quantification.

### Analytical antibody phospho-specificity evaluation

To indirectly assess the phospho-specificity of the ATM serine-1981 and γH2AX antibodies, membranes were incubated with 1000 U of lambda protein phosphatase (λPP) for 30 min at 37°C. Membranes were rinsed twice with TBS and processed for protein detection and signal quantitation. Incubation with λPP reduced ATM serine-1981 phosphorylation signal intensity to undetectable levels (data not shown). The γH2AX signal was detectable in the 2 Gy specimen on the λPP-treated membrane, but it was reduced to 7% of the γH2AX signal on the non-λPP- treated membrane. This demonstrates that both antibodies are indeed phospho-specific.

### Intra-Gel/Immunoblot imprecision estimations

To estimate intra-assay reproducibility, protein extracts prepared from human PBMCs obtained from control or 2 Gy irradiated whole blood were assayed in octuplicate on separate gels. To minimize possible variability associated with gel loading position, each gel had the control loaded in even numbered lanes and 2 Gy samples were loaded in an alternating fashion in the odd numbered lanes across the gel. Table 1 shows the estimated imprecision for ATM and H2AX detection in PBMCs from two subjects (A and B).

**Table 1:** Intra-assay Imprecision

Sample ID	Analyte	Mean Signal Intensity (RFU × 10^5)^	cv (%)	n	Ratio (phospho/pan protein)	cv (phospho/pan protein) (%)	Fold-Activation
Control A	phospho-ATM	4.64 ± 0.24	5.1%	8	0.07 ± 0.003	4.7%	
Control B	phospho-ATM	3.49 ± 0.17	4.9%	8	0.12 ± 0.008	6.2%	
Control A	pan-ATM	64.3 ± 1.16	1.8%	8			
Control B	pan-ATM	28.2 ± 1.26	4.5%	8			
							
2 Gy IR A	phospho-ATM	47.9 ± 1.94	4.0%	8	0.7 ± 0.036	4.9%	10.38 ± 0.81
2 Gy IR B	phospho-ATM	57.8 ± 0.92	1.6%	8	1.26 ± 0.035	2.8%	10.21 ± 0.80
2 Gy IR A	pan-ATM	64.2 ± 2.09	3.3%	8			
2 Gy IR B	pan-ATM	45.9 ± 1.39	3.0%	8			
							
Control A	γH2AX	45.7 ± 0.69	1.5%	8	8.37 ± 0.50	6.0%	
Control B	γH2AX	20.1 ± 1.20	6.0%	8	8.21 ± 0.87	10.6%	
Control A	pan-H2AX	5.42 ± 0.29	5.3%	8			
Control B	pan-H2AX	2.47 ± 0.21	8.6%	8			
							
2 Gy IR A	γH2AX	165 ± 2.70	1.6%	8	29.60 ± 0.83	2.8%	3.55 ± 0.19
2 Gy IR B	γH2AX	163 ± 6.59	4.0%	8	29.91 ± 1.48	5.0%	3.68 ± 0.41
2 Gy IR A	pan-H2AX	5.58 ± 0.18	3.2%	8			
2 Gy IR B	pan-H2AX	5.47 ± 0.18	3.3%	8			

In all cases, the imprecision (cv%) was acceptably less than 10%. The observed fold increase in ATM phosphorylation was similar between subjects (10.4-fold and 10.2-fold for subjects A and B, respectively). The observed increase in γH2AX was also similar between subjects (3.6-fold and 3.7- fold, respectively). A Z' value provides a measure of assay quality taking account of both the signal intensity and assay variability [19]. Assays with Z' values between 0.5 and 1 are considered excellent. Selecting the 2 Gy irradiated specimen as the positive control and the unirradiated specimen as the negative control, the ATM signal ratio yields a Z' value of 0.82 and 0.89 for subjects A and B, respectively. The H2AX signal ratio generated Z'values of 0.81 and 0.67 for subjects A and B, respectively.

### Dilutional linearity determinations

**Figure 2:**
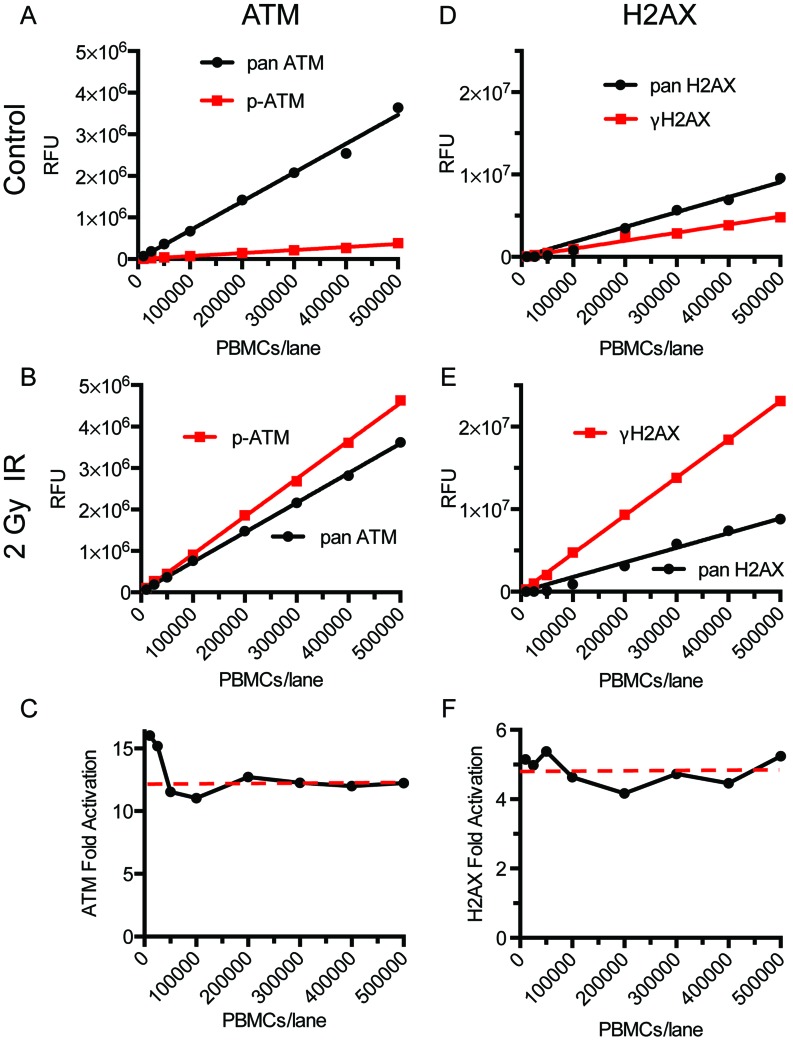
Effect of PBMC lysate dilution on protein quantitation Cellular lysates, prepared from lllltreated **(A,D)** and 2 Gy irradiated PBMCs **(B,E,)** from subject B were serially diluted and processed by ATM **(A,B)** and H2AX **(C,D)** immunoblot analysis. Fold activation ofATM (C) and H2AX (F) were analyzed as a function ofPBMCs per gel lane.

To assess relative analytical accuracy, estimate analytical sensitivity, and establish the range of cell densities that produce linear immunoblot signals, two irradiated PBMC specimens and their matched control specimens were fractionally diluted 50-fold, in seven discrete steps (2 × 10^7^ to 4 ×10^5^ cells/mL), processed in an identical manner, and analyzed for ATM and H2AX signal intensities. This analysis was performed in duplicate using a separate gel for each specimen type for each subject (a total of four gels were run). The multiplexed ATM immunoblot from subject B showed excellent dilutional linearity across the entire 50-fold range of cell concentrations assessed for both control and irradiated specimens (Figure 2A, 2B). Linear regression correlation coefficients (r) were >0.999 for all ATM dilution curves. The ATM phosphorylation/pan ATM ratio for subject A was consistent across all dilutions with a coefficient of variation (cv) of 21% and 9% (n = 8) for the control and 2 Gy specimens, respectively. Likewise, the ATM phosphorylation/pan ATM ratio for subject C showed cv of 9% and 10% (n = 8) for the control and 2 Gy specimens, respectively (data not shown). To determine the fold activation of ATM following DNA damage, the ratio of ATM phosphorylation/pan ATM after irradiation was normalized to the ratio obtained in control unirradiated PBMC lysates. The mean increase in ATM phosphorylation was 12.8-fold and had an acceptable associated cv (14%). However, the two highest dilutions showed a deviation from all previous dilutions (Figure 2C). The increase in ATM phosphorylation in subject C was significantly greater (30.7-fold) with a cv of 9%. Thus, an acceptable lower cell limit is 50,000 PBMCs/lane for detection of an increase in ATM phosphorylation.

The corresponding multiplexed H2AX immunoblot showed highly acceptable dilutional linearity (Figure 2D, 2E). Linear regression correlation coefficients (r) were >0.987 for all H2AX dilution curves. However, the γH2AX/pan H2AX ratio was only uniform to 200,000 PBMCs/lane, with cv of 15% and 26% for the control and the 2 Gy specimen, respectively. Higher dilutions resulted in significantly more variable cv values due to lower RFU. The calculated mean fold activation of H2AX following 2 Gy is 4.8-fold for subject B (Figure 2F) and 4.1-fold for subject C. However, given the higher variability at lower cell dilutions, an acceptable cell limit for detection of increased γH2AX is 200,000 PBMCs/lane.

### Specimen stability study

**Figure 3:**
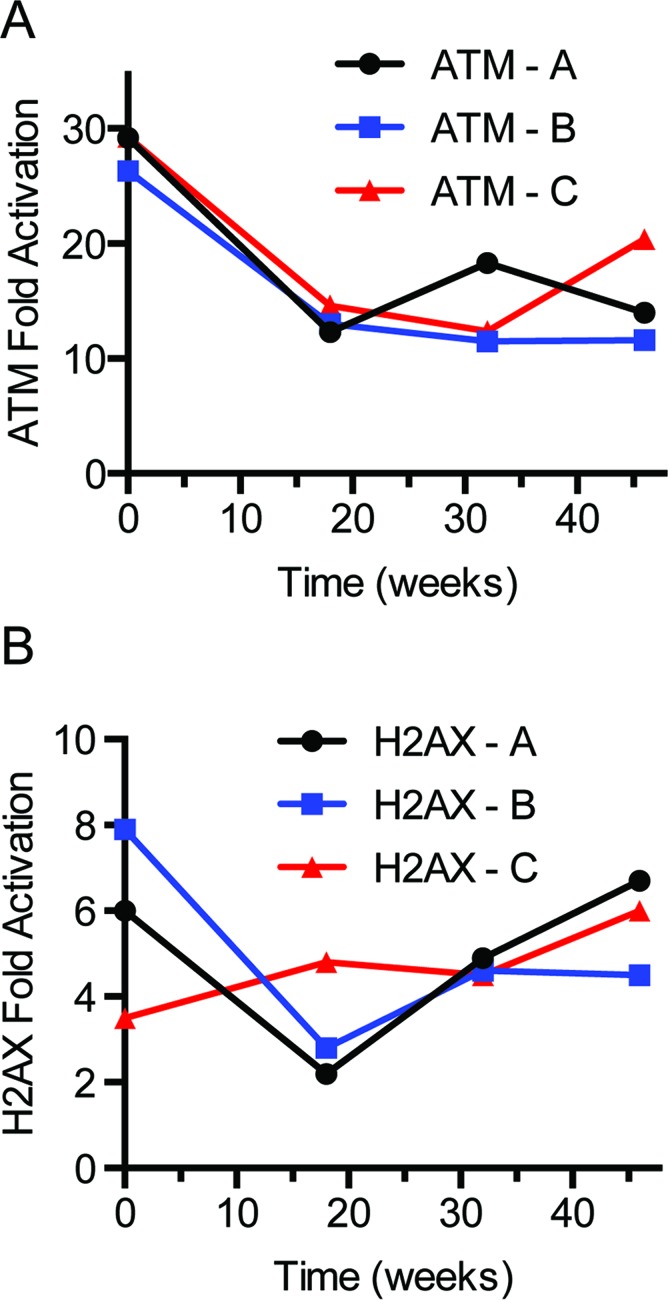
Effect of storage time on protein fold activation Blood tubes from three subjects were 2 Gy irradiated and PBMCs were isolated and stored at −70°C. Immediately after irradiation and over 46 weeks, PBMC pellets were analyzed for ATM (**A**) and H2AX activation (**B**).

An important parameter that permits measurement of a biomarker clinically is the stability of the analyte upon long-term storage. Whole blood was obtained from 3 healthy subjects (2 male; 1 female) and irradiated with 2 Gy. PBMCs were harvested and stored at −70°C until analysis. The increase in ATM and H2AX phosphorylation was measured immediately after irradiation and up to 46 weeks stored at −70°C. The mean fold increase in ATM phosphorylation among the 3 subjects was 18-fold (Figure 3A). The mean fold activation for H2AX was 5-fold (Figure 3B). The observed variation for both analytes is expected and acceptable for a fit-for-purpose assay. This is an ongoing study with plans to test stability over 2 years.

### ATM and H2AX phosphorylation induction in PBMCs exposed to chemotherapeutic agents *in vitro*

**Figure 4:**
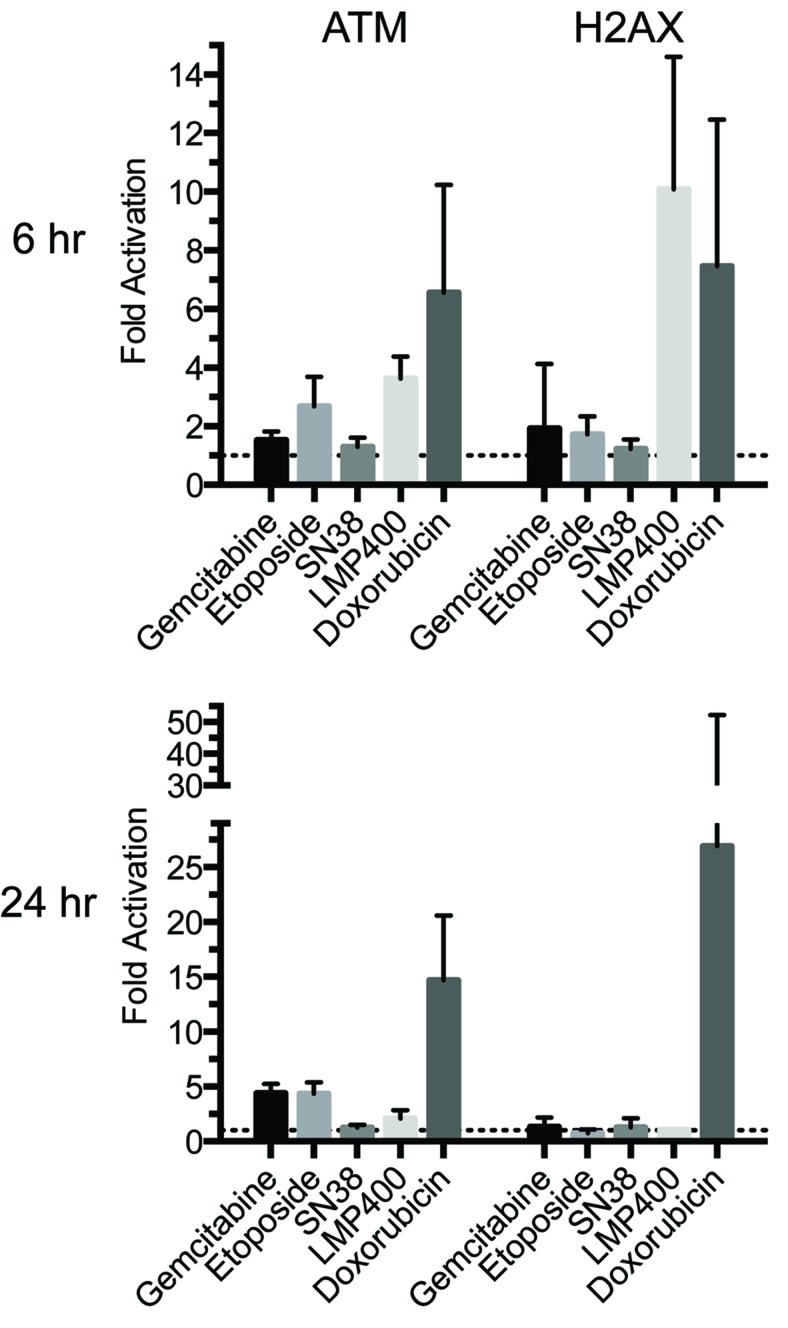
Effect of various chemotherapeutic agents on DNA damage response in PBMCs PBMCs were incubated with the indicated agents for 6 and 24 hr. Cells were harvested and processed for immunoblot analysis. The values represent the mean ± S.D. from duplicate measurements from three independent experiments.

To assess increased ATM and H2AX phosphorylation in PBMCs exposed to chemotherapeutic agents *in vitro*, PBMCs were isolated from a single subject, plated at ~8 × 106 cells in 6-well plates and exposed to either vehicle, gemcitabine (20 μM), etoposide (3 μM), SN38 (66 nM), LMP400 (2 μM; Dr. Jan Beumer, personal communication), or doxorubicin (1.7 μM) for up to 24 h at 37°C. These drug concentrations are all obtainable in human plasma [20-23]. These DNA- damaging chemotherapeutic agents increased ATM and H2AX phosphorylation in PBMCs *in vitro* at 6 h, with LMP400 and doxorubicin inducing the greatest ATM serine-1981 phosphorylation (3.63-fold and 6.56-fold, respectively) and LMP400 and doxorubicin inducing the greatest γH2AX at this time point (10.1-fold and 7.46-fold, respectively) (Figure 4). Doxorubicin increased ATM and H2AX phosphorylation in PBMCs *in vitro* still further at 24 h (14.70-fold and 26.93-fold, respectively). At 24 h, LMP400-induced ATM phosphorylation decreased to 2-fold while γH2AX levels returned to basal levels. SN38 had little to no effect on ATM and H2AX phosphorylation at either time point. Of note, both gemcitabine and etoposide treatment at 24 h increased ATM phosphorylation (4.4-fold for both agents).

### ATM and H2AX phosphorylation in PBMCs exposed to doxorubicin and DNA repair inhibitors *in vitro*

**Figure 5:**
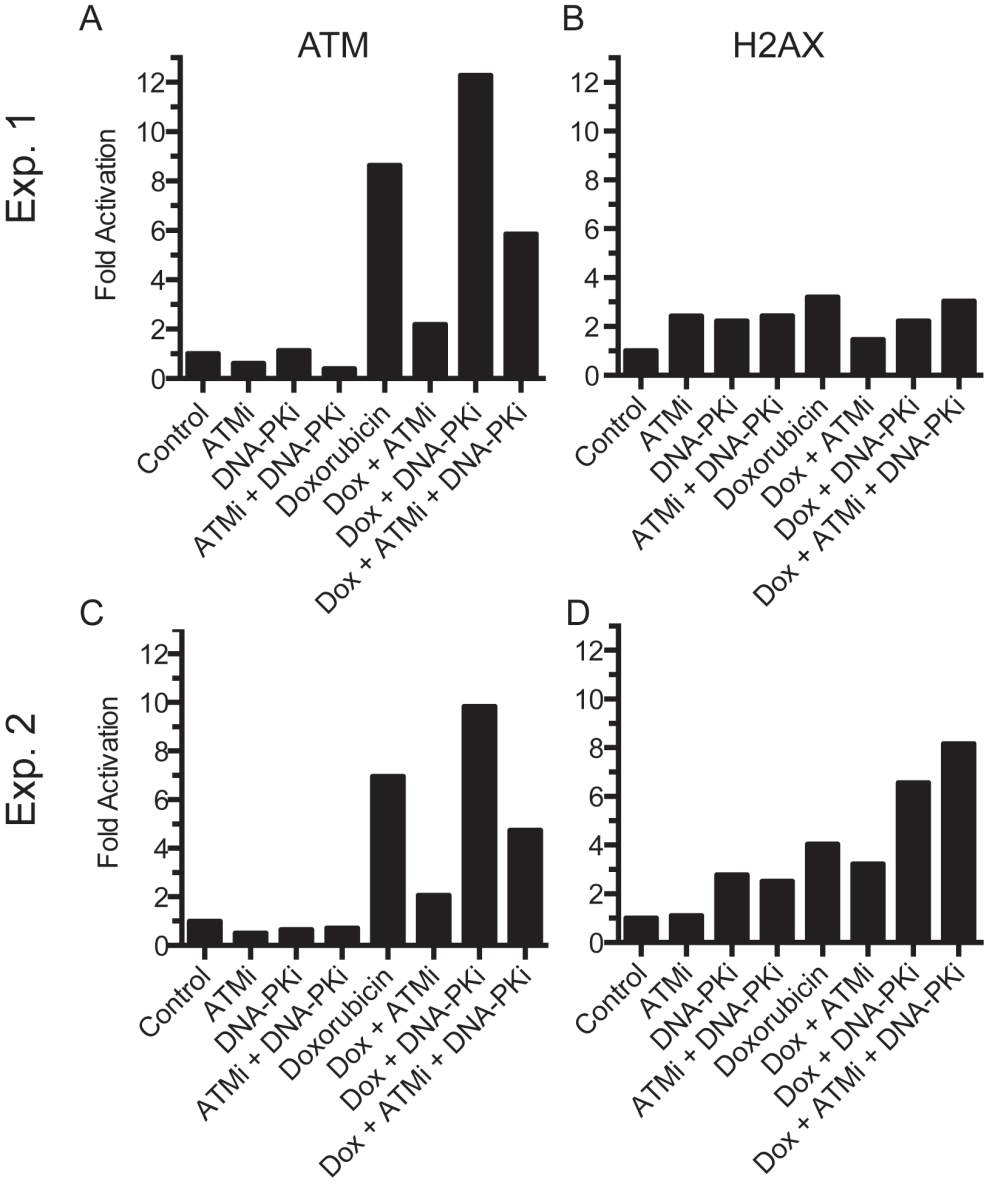
Effect of doxorubicin in combination with kinase inhibitors on DNA damage response in PBMCs PBMCs were incubated with kinase inhibitors in the presence or absence of doxorubicin (1.7 uM) for 6 hr. Cells were harvested and processed for immunoblot analysis. Data from two separate experiments is shown.

To explore the DNA damage signaling to ATM and H2AX in PBMCs treated with doxorubicin *in vitro*, we used KU55933 and NU7441, pharmacologic inhibitors of ATM kinase and DNA-PK kinase activities, respectively [4,8]. Doxorubicin-induced ATM serine-1981 phosphorylation was dramatically inhibited by ATM kinase inhibitor KU55933 (8.64- vs. 2.19-fold) suggesting that doxombicin induces DSBs that induce ATM kinase phosphorylation directly (Figure 5A). Concurrent treatment with doxombicin and inhibition of DNA-PK using NU7441 increased ATM serine-1981 phosphorylation still further (12.28-fold) and this was only partially decreased by ATM kinase inhibitor (5.85- fold). Similarly, H2AX phosphorylation remained elevated upon addition of both kinase inhibitors (Figure 5B). This suggested that a third kinase phosphorylates ATM serine-1981 when ATM and DNA-PK kinase activities are inhibited.

### ATR and SMG1 expression in PBMCs

**Figure 6:**
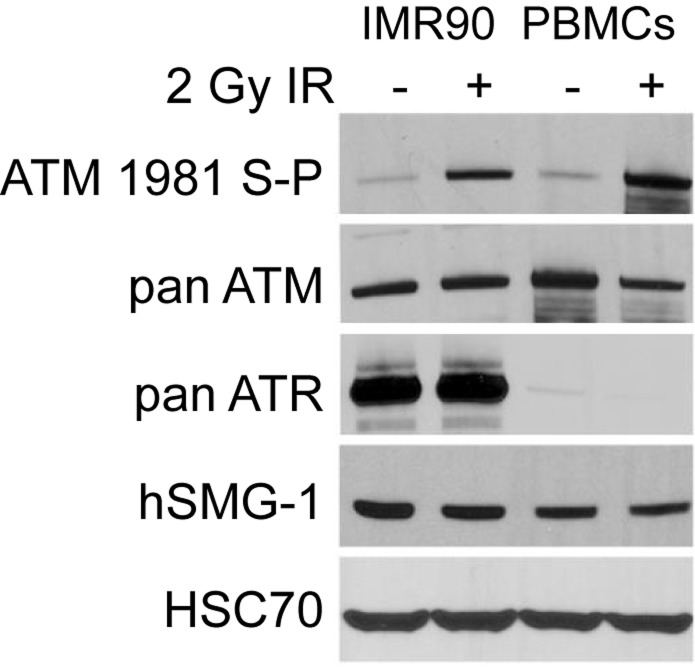
Protein expression after irradiation PBMCs and IMR90 lung fibroblasts were irradiated and processed for immunoblot analysis.

ATR phosphorylates ATM serine-1981 in cells exposed to DNA damaging modalities that induce stalled replication forks including UV and hydroxyurea (HU) [17]. However, there are conflicting reports of the expression of ATR in PBMCs. While several reports document an absence of detectable expression of ATR in PBMCs [24,25], a recent report suggests that ATR is detectable in this cell type [26]. To determine the expression of ATR in PBMCs, we generated cell extracts from IMR90 primary human lung fibroblasts and PBMCs that had been stored, frozen at −70°C for 50 weeks. These extracts were resolved and immunoblotted for ATM serine-1981 phosphorylation, pan-ATM, pan-ATR, pan-SMG1 and control HSC70. ATM, ATR, DNA-PK and hSMG-1 are related phosphatidylinositol 3-kinase-related kinases (PIKKs) and hSMG-1 is essential for the degradation of mRNA molecules containing premature stop codons that may give rise to dominant-inhibitory truncated proteins [27]. hSMG1 is also implicated in DNA damage signaling [28]. While ATR is barely detectable in PBMCs, ATM and hSMG-1 are expressed in PBMCs and IMR90 primary fibroblasts at similar levels (Figure 6).

### DNA damage signaling in patients treated with doxorubicin for sarcoma *in vivo*

**Figure 7:**
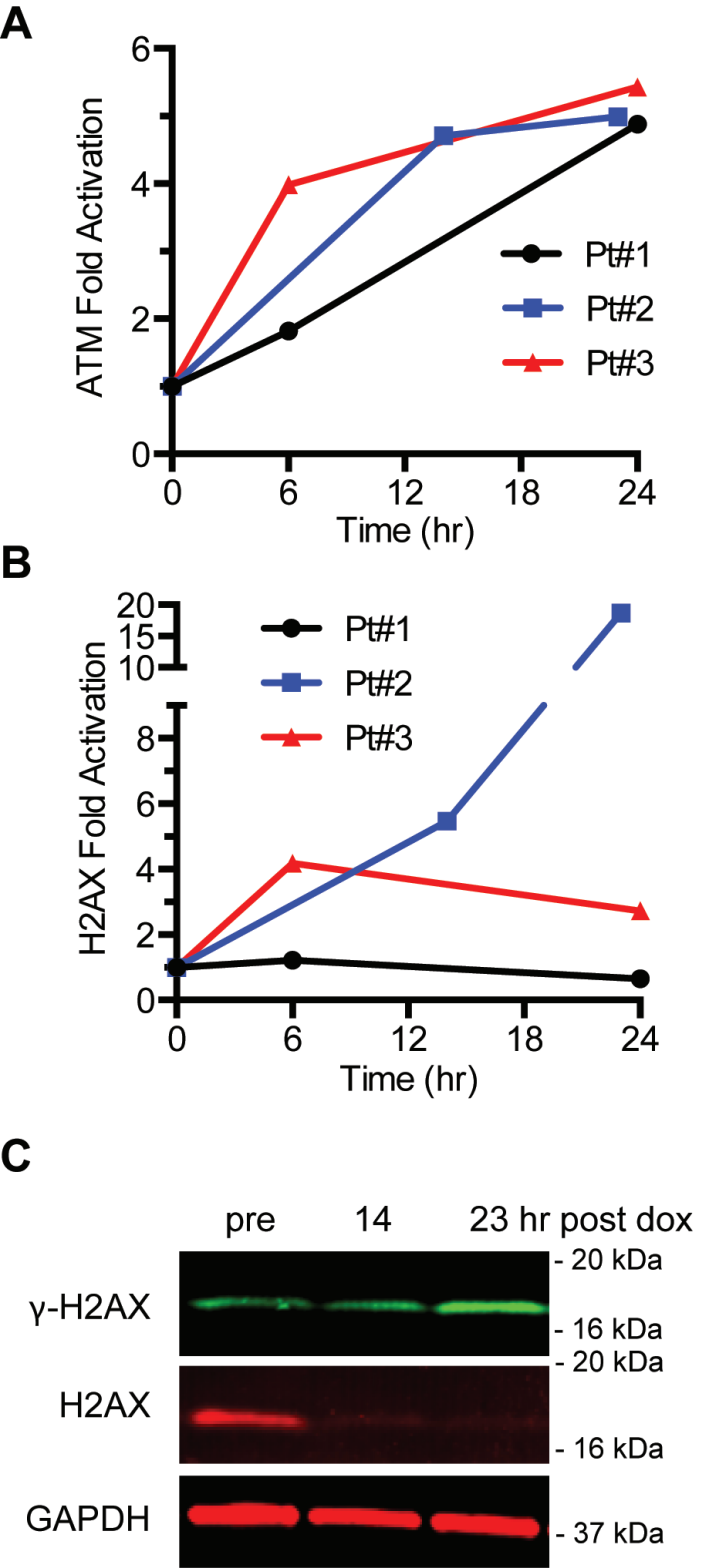
DNA damage response in patient PBMCs PBMCs were obtained from three patients treated with doxorubicin/ifosfamide. Samples were processed for immunoblot analysis for detection of ATM (**A**) and H2AX activation (**B**). (**C**) Image of H2AX immunoblots in PBMCs from patient#2.

To determine whether ATM and H2AX phosphorylation are increased in patients treated with an agent that induces ATM and H2AX phosphorylation *in vitro*, blood was drawn from three patients undergoing treatment for sarcoma before and at either 6, 14, and 24 h after infusion with the first cycle of doxorubicin (75 mg/m2) followed by ifosfamide (3 g/m2). ATM serine 1981 phosphorylation was induced after 6 h and continued to increase at 24 h in all three patients (Figure 7A). While showing significantly increased ATM serine 1981 phosphorylation, patient #1 showed no induction of γH2AX (Figure 7B). In patient #3, γH2AX decreased with time whereas ATM serine 1981 phosphorylation increased. In patient #2, γH2AX was induced very dramatically at 24 h (18.7-fold). However, this result in patient #2 is a consequence of a significantly decreased level of pan H2AX signal at later times resulting in a high γH2AX/pan H2AX ratio (Figure 7C).

## DISCUSSION

Here we document the pre-clinical analytical, and fit-for-purpose validation of a quasi-quantitative dual multiplexed immunoblot method to simultaneously analyze ATM and H2AX phosphorylation in human peripheral blood mononuclear cells (PBMCs). The major accomplishments of this work are: (1) the validation of a novel methodology to detect increased ATM and H2AX phosphorylation in patients receiving chemotherapy; (2) the recognition of the logistical and ethical advantage imparted through the use of PBMCs as a surrogate tumor tissue specimen, thus, minimizing the need for more invasive specimen collection procedures; (3) the effective reduction of the required sample volume by multiplexing all four protein targets onto a single gel; (4) the improved technical efficiency and increased analytical confidence by the elimination of the need to strip and re-probe membranes; (5) the enhanced analytical accuracy achieved by using ratio-metric analyses which normalizes signal intensity and corrects for loading and sampling errors; (6) and, the use of commercially available critical reagents, thus, making this procedure readily accessible for the entire scientific community.

Many of technical accomplishments in this quasi- quantitative dual multiplexed immunoblot method can be attributed to the use of near-infrared (NIR) fluorophores which have a number of advantages over conventional chemiluminescence technologies. NIR fluorophores provide analytical sensitivity that is at least equal to chemiluminescence [29,30]. NIR fluorochromes also generate static signals that can be measured with superior accuracy and precision over the dynamic signals generated by chemiluminescence which quickly fades over time. Furthermore, membranes and biomolecules show little autofluorescence in the longer NIR wavelength region, dramatically lowering background and significantly increasing the associated signal-to-noise ratio. Finally, the simultaneous generation of two independent NIR signals, characteristic of multiplexed immunoblots, enables a ratio-metric approach to data analysis. Expressing signals as a ratio corrects for loading and sampling errors, thus greatly increasing the accuracy of and confidence in the resultant data [13]. Our methodology delivers ATM serine-1981 phosphorylation as a proven analytically sensitive, specific and robust PD biomarker for clinical trials.

Implementations of reliable techniques that measure functional pharmacokinetic (PK) and PD biomarkers are essential for rational and efficient development of new molecular cancer therapies. Early on in the course of treatment, an ideal PD biomarker would be able to determine if an agent is reaching its target and acting as intended, thus facilitating proof-of-concept demonstrations of target engagement. If PD biomarkers obtain greater specificity and reliability, establishing a minimally effective dose in phase I trials instead of a maximum tolerated dose may become feasible. Further, applying PK/PD biomarker information at the beginning at the outset of treatment may aid in identifying the most appropriate patients, and lead to a more personalized approach to the delivery and dosage of a single drug or combinations of drugs.

ATM kinase is activated at DSBs through intermolecular autophosphorylation on serine-1981 and we have reported previously, using the method documented in detail here, that ATM serine-1981 phosphorylation is increased in the PBMCs of patients receiving stereotactic radiotherapy [15,31]. As such, ATM serine-1981 phosphorylation is an excellent PD biomarker for target engagement by pharmacologic ATM kinase inhibitors. Our finding that ATR expression in PBMCs is at the limits of detection initially suggested that PBMCs may be an ideal cell in which target engagement by ATM kinase inhibitors can be examined, as ATR kinase-dependent phosphorylation of ATM serine-1981 is likely to be minimal or absent [17].

Our finding that ATM serine-1981 phosphorylation is high in PBMCs treated with doxorubicin, ATM kinase inhibitor and DNA-PK inhibitor, shows that a third kinase phosphorylates this site in PBMCs. One candidate is hSMG-1, a fourth PIKK that, along with ATM, ATR and DNA-PK, has been implicated in DNA damage responses [28]. To our knowledge, the expression of hSMG-1 has not been investigated in PBMCs previously. We show here that hSMG-1 is similarly expressed in IMR90 primary fibroblasts and PBMCs rendering it a candidate for ATM serine-1981 phosphorylation in PBMCs. A second candidate kinase for the ATM phosphorylation observed in PBMCs is I kappa B kinase/nuclear factor kappa B (IKK/NF-κB) which has been reported to directly phosphorylate ATM serine-1981 [32]. This illustrates the complexity of the DNA damage response system and the challenges in identifying a PD biomarker for target engagement therein that is unambiguous.

Our finding that ATM serine-1981 phosphorylation is increased in the PBMCs of patients immediately following infusion with doxorubicin/ifosfamide and our previous report that ATM serine-1981 phosphorylation is increased in patients receiving stereotactic radiotherapy demonstrate that this phosphorylation is an excellent PD biomarker for radiation and chemotherapy-induced DNA damage. While it is generally classified as a topoisomerase II-stabilizing drug that induces DSBs, doxorubicin can intercalate DNA and generate reactive oxygen species and it has been shown previously that the hydroxyl radical scavenger, N-acetylcysteine, can attenuate doxorubicin-induced ATM kinase activity [33]. Thus, hydroxyl radicals may be the principal inducers of DSBs and ATM serine-1981 phosphorylation in both irradiated and doxorubicin-treated cells. This may explain our observation that doxorubicin induces the greatest ATM serine-1981 phosphorylation in non-replicating PBMCs cultured *in vitro*.

ATM serine-1981 phosphorylation may be a more selective PD biomarker for DSBs than γH2AX. Previous reports suggest that ATM serine-1981 phosphorylation increases with dose of γ-rays in a linear manner until the maximal stoichiometry of the ATM phosphorylation is reached [15,16]. While γH2AX also increases with dose of γ-rays in a linear manner to a point, above some threshold of base, single-strand breaks or DSBs that is not understood, pan-nuclear γH2AX is suddenly induced [13,14]. Pan-nuclear γH2AX is reversible and therefore not restricted to apoptotic cells [14]. This non-linearity with DSBs may be misleading in immunoblotting analyses where γH2AX foci located at DSBs are not distinguished from pan-nuclear γH2AX staining. While the ATM serine-1981 phosphorylation induced in three patients treated with doxorubicin is similar, three different patterns of γH2AX induction are seen over time. In one patient, γH2AX is not induced. In another patient γH2AX is induced at 6 h and then reduced by 24 h. Finally, in a third patient γH2AX is induced at 12 h and then induced dramatically higher at 24 h. It is possible that this dramatic increase in γH2AX at 24 h that is not mirrored by ATM serine-1981 phosphorylation is a manifestation of pan-nuclear γH2AX. Further studies are needed to address this possibility. The quasi-quantitative dual multiplexed immunoblot method to simultaneously analyze ATM and H2AX phosphorylation in PBMCS that we document here is currently being used as a PD marker for phase I clinical trials at the University of Pittsburgh Cancer Institute.

## MATERIALS AND METHODS

### Subjects

All studies were approved by the UPMC Health System/University of Pittsburgh Institutional Review Board (IRB). Written informed consent was obtained from each subject.

### Materials

The following reagents were used for tissue culture: IMR90 human lung fibroblast cells, American Type Culture Collection (Manassas, VA); RPMI-1640, Life Technologies (Carlsbad, CA); 10% fetal bovine serum, Gemini Bio- Products (West Sacramento, CA); MycoAlert Mycoplasma detection assay, Cambrex BioScience (Rockland, ME).

The following reagents were used for electrophoresis: glycine, Tween 20, Tris, 10x Tris/Glycine/SDS electrophoresis running buffer, precision plus protein standards (10 – 250 kDa), Tris-HCL and 4-15% TGX Criterion^™^ precast (18-well) gels from Bio-Rad Laboratories (Hercules, CA).

anti-human ATM serine-1981 phosphorylation antibody, clone EP1890Y, Abcam (Cambridge, MA); purified mouse monoclonal anti-human/mouse pan-ATM antibody, clone 2C1, GeneTex (Irvine, CA); biotinylated rabbit monoclonal anti-human/mouse/rat/monkey histone H2AX serine-139 phosphorylation, clone 20E3, Cell Signaling Technology (Danvers, MA); mouse monoclonal anti-human/mouse/rat histone H2AX antibody, clone 322105, R&D Systems (Minneapolis, MN); rabbit monoclonal anti-human/rabbit/mouse ATR, clone E1S3S, Cell Signaling Technology (Danvers, MA); SMG-1, (Q25), Cell Signaling Technology (Danvers, MA); IRDye 800CW conjugated goat anti-rabbit, and IRDye 680RD conjugated goat anti-mouse LI-COR Biotechnology-US (Lincoln, NE). Additional immunoblot reagents including Odyssey® blocking buffer and two-color molecular weight markers (7-250 kDa) were purchased from LI-COR Biotechnology-US (Lincoln, NE).

Other regents used: DyLight 800 conjugated neutravidin and Pierce® BCA protein assay kit, Thermo Scientific, Inc. (Rockford, IL), phosphatase and protease inhibitor cocktail tablets, Roche Diagnostics (Indianapolis, IN); lambda protein phosphatase (λPP), New England BioLabs Inc. (Ipswich, MA); Plasma Lyte-A USP, Baxter (Deerfield, IL). Gemcitabine, etoposide, SN38, and doxorubicin were obtained from Sigma (St. Louis, MO). LMP400 was provided by Dr. Mark Cushman (Purdue University). ATM kinase inhibitor KU55933 and DNA-PK kinase inhibitor NU7441 were a kind gift of Mark O'Connor, PhD (AstraZeneca, Macclesfield, UK).

### Buffer formulations

Dithiothreitol (DTT)-Modified Laemmli buffer: 50 mM Tris-HCl (pH 6.8) containing 2% (w/v) SDS, 10% (v/v) glycerol, 200 mM DTT and 0.002% (w/v) bromophenol blue supplemented with protease and phosphatase inhibitor cocktails. Towbin buffer: [34]; 25 mM Tris, 192 mM glycine, (~pH 8.3) with 20% (v/v) methanol. 0.5x Odyssey® blocking buffer (OBB): Odyssey® blocking buffer mixed 1:1 with TBS (v/v). OBB-T: OBB containing 0.1% (v/v) Tween-20.

### Blood collection and PBMC isolation

Human blood (~8.0 mL/tube) was drawn into Cell Preparation Tubes (CPT) (Becton Dickinson, Franklin Lakes, NJ). Where indicated, whole blood was gamma-irradiated in a Shepherd Mark I Model 68 [137Cs] irradiator (J.L. Shepherd & Associates) at a dose rate of 71.1 Rad/min. PBMCs were isolated by centrifugation at 1500 ×g for 30 min at room temperature (RT). The PBMC layer was transferred to a new 15 mL polypropylene conical tube and washed with three to four volumes of Plasma-Lyte A USP. The cells were pelleted by centrifugation (430 ×g for 10 min at RT). The PBMCs were resuspended in 3.0 mL of was removed and used to estimate PBMC density with a Beckman Coulter Z1 particle counter before adding another wash volume of 10 mL of Plasma-Lyte A. After re-pelleting, aspiration and disposal of the supernatant, the PBMC density was adjusted to 10^7^ cells/mL in Plasma-Lyte A. Single-use aliquots of predetermined volumes were then transferred into new 2.0 mL micro-tubes. PBMCs were recovered as cell pellets by centrifugation (10,000 × g for one min at RT), and flash-frozen in a dry ice/ethanol bath before storage at −70°C until use. To preserve specimen integrity, all CPT tubes were processed within three hours of collection.

### Lysate/extract preparation of human PBMCs

Frozen PBMC pellets were resuspended at 2 × 10^7^ cells/mL in DTT-modified Laemmli buffer freshly supplemented with protease and phosphatase inhibitor cocktails, vortexed and pulsed sonicated at 100% amplitude, for three cycles of 2.5 s on and 10 s off, in an ice-cold cup- horn sonicator (Cole-Parmer, Vernon Hills, IL). Lysates were heated to 95°C for seven min, vortexed and chilled briefly on ice. After centrifugation at 10,000 × g for 5 min at RT, supernatants were transferred into new 1.5 mL micro-tubes.

### Specimen storage stability

Eight CPT (8 mL/CPT) tubes per person were collected from two normal male and one female subjects. Four CPTs from each volunteer were irradiated with a single exposure of 2.0 Gy of ionizing radiation. The remaining four unirradiated CPTs served as a baseline control for each subject. PBMC pellets (1.5 ×10^6^ cells/pellet) were isolated as described above and then stored at –70°C. To evaluate specimen stability, baseline and 2.0 Gy pellets from each volunteer were assayed in concert on the same gel, the next day (time = 0), at 18, 32, and 46 weeks, and at 14 week intervals thereafter.

### Detailed immunoblot procedure

To avoid bias in protein levels due to possible plasma protein contamination, the PBMC samples were normalized by cell number [35]. Aliquots of 25 μL (5 ×10^5^ cells) were loaded per well onto 4-15% TGX Criterion^™^ 18- well precast gels in a Criterion^™^ vertical midi-format electrophoresis cell (Bio-Rad Laboratories, Hercules, CA), containing 0.5 L/gel of 1x Tris/Glycine/SDS running buffer. Additionally, 7 μL aliquots of a commercially available mixture of prestained protein standards were loaded into the first, middle, and last well. Proteins were fractionated under a constant 200 V applied for ~40 min. Upon SDS-PAGE completion, gels were equilibrated in cold Towbin buffer for 10 min. Resolved proteins were electrophoretically transferred out of the PAGE gel and onto 0.45 μm Immobilon-FL polyvinylidene difluoride (PVDF) transfer membranes (Millipore, Billerica, MA) with 100 V applied for 1.5 h in a Criterion blotter transfer tank (Bio-Rad Laboratories, Hercules, CA) filled with cold Towbin buffer. To maintain chilled transfer conditions, the tank's ice packs were changed at 45 min intervals.

### Membrane blocking and immunoprobing

Following electroelution transfer, membranes were rinsed with water and incubated in 0.5x Odyssey® blocking buffer (20 mL/membrane) for one h at RT with gentle agitation. Membranes were cut horizontally into strips. The top membrane strip (above 75 kDa) was immunoprobed with a monoclonal rabbit anti-phosphoserine1981 ATM and a purified mouse monoclonal anti-pan-ATM antibody mixture diluted to 1:500 and 1:2500, respectively, in 0.5x Odyssey® blocking buffer containing 0.1% Tween 20 (OBB-T). Likewise, the bottom membrane strip (below 25 kDa) was simultaneously probed with an antibody mixture consisting of a biotinylated monoclonal rabbit anti-γH2AX antibody and a mouse monoclonal anti-H2AX antibody, each diluted to 1:1000 in OBB-T. Strips were separately incubated overnight at 4°C with gentle agitation. The membrane strips were rinsed with TBS, washed thrice with 50 mL of TBS-T, for periods of 10 min each. The ATM membrane strip was immunoprobed with a pair of highly cross-absorbed anti-IgG detection antibodies specifically matched to the two different primary ATM antibody host species. These detection antibodies, a goat anti-rabbit IgG and a goat anti-mouse IgG antibody were labeled with NIR fluorochrome dyes (800 and 700 nm) and diluted 1:4000 and 1:5000, respectively. The H2AX membrane strip was similarly probed with both antibodies diluted 1:5000 in addition to streptavidin conjugated to an 800 nm fluorophore diluted 1:10000. All detection reagents were prepared in OBB-T containing 0.02% (w/v) SDS. The strips were incubated in the dark for 1 h at RT with gentle agitation. Membranes were washed thrice, each for five min in 50 mL of TBS-T and twice in 50 mL of TBS for 10 min. All five washes occurred undulating in the dark at RT. Before proceeding, membranes were dried at RT for at least one h in the dark.

### Immunoblot imaging and signal quantification

Dry membrane strips were visualized using an Odyssey® CLx infrared imaging system (model 9120) (LI-COR Biosciences-US, Lincoln, NE). This instrument employs two NIR lasers and detectors to image fluorescent emissions at 710 and 805 nm concurrently. To ensure the highest possible intensity settings, each individual ATM or H2AX membrane strip was scanned manually. However, for gels assayed in tandem, the analogous pairs of ATM or H2AX strips were each scanned in concert. Signal quantification was performed utilizing the version 2.1 Image Studio software (LI-COR). Shapes were drawn around bands of interest, and the signal was calculated as the sum of the individual pixel intensity values (Total) for the selected shape minus the product of the average intensity values of the pixels in the background (Bkg) and the total number of pixels enclosed by the shape (Area). Thus, Signal = Total – (Bkg × Area). The selected background value applied for each individual shape was the median of three pixels above and below the shape. The ratio of the phosphorylated to pan-protein signal was used to correct for loading variations between lanes.

Conventional immunoblotting and chemiluminescence on PBMCs and IMR90 cells was performed as described previously [16].

### Statistical analysis

Unless stated otherwise, data are presented as mean ± standard deviation (SD). For paired Student's t-tests and one- way ANOVA F-tests, *P* < 0.05 was considered significant. The Z' value [19] provides a measure of assay quality taking account of both the signal intensity and assay variability. Z' = 1 – (3 × Positive Control SD + 3 × Negative Control SD) ÷(mean Positive Control – mean Negative Control). Z' values of 1 are perfect, values > 0.5 are considered acceptable. Linear regression analysis was performed using Microsoft Office Excel 2007. Linear regression coefficients (r) > 0.9 are considered acceptable.
